# Comparative Analysis of Four Complete Mitochondrial Genomes of Epinephelidae (Perciformes)

**DOI:** 10.3390/genes13040660

**Published:** 2022-04-08

**Authors:** Chen Wang, Peiyuan Ye, Min Liu, Yue Zhang, Haiqing Feng, Jingyu Liu, Haolang Zhou, Junjie Wang, Xiao Chen

**Affiliations:** 1 College of Marine Sciences, South China Agriculture University, Guangzhou 510642, China; wangchen2971@163.com (C.W.); cuiluoshi1998@163.com (P.Y.); zzyue01@163.com (Y.Z.); fhqing1@163.com (H.F.); scauliujingyu@163.com (J.L.); 2 College of Marine Sciences, Shanghai Ocean University, Shanghai 201306, China; 3 State Key Laboratory of Marine Environmental Science, College of Ocean and Earth Sciences, Xiamen University, Xiamen 361012, China; minliuxm@xmu.edu.cn; 4 Guangxi Mangrove Research Center, Beihai 536000, China; zhouhaolang@sina.com; 5 Guangzhou Key Laboratory of Subtropical Biodiversity and Biomonitoring, School of Life Sciences, South China Normal University, Guangzhou 510631, China

**Keywords:** groupers, Epinephelidae, mitogenome, purify selection, phylogenetic analyses

## Abstract

Groupers are commercial, mainly reef-associated fishes, classified in the family Epinephelidae (Perciformes). This study first sequenced the complete mitogenomes of *Cephalopholis leopardus*, *Cephalopholis spiloparaea*, *Epinephelus amblycephalus*, and *Epinephelus hexagonatus*. The lengths of the four Epinephelidae mitogenomes ranged from 16,585 base pair (bp) to 16,872 bp with the typical gene order. All tRNA genes had a typical cloverleaf structure, except the tRNA-*Ser* (AGY) gene which was lacking the entire dihydrouridine arm. The ratio of nonsynonymous substitution (Ka) and synonymous substitution (Ks) indicated that four groupers were suffering a purifying selection. Phylogenetic relationships were reconstructed by Bayesian inference (BI) and maximum likelihood (ML) methods based on all mitogenomic data of 41 groupers and 2 outgroups. The identical topologies result with high support values showed that *Cephalopholis* and *Epinephelus* are not monophyletic genera. *Anyperodon* and *Cromileptes* clustered to *Epinephelus*. *Aethaloperca rogaa* and *Cephalopholis argus* assembled a clad. *Cephalopholis leopardus*, *C. spiloparaea*, and *Cephalopholis miniata* are also in a clade. *Epinephelus* *hexagonatus* is close to *Epinephelus tauvina* and *Epinephelus merra*, and *E. amblycephalus* is a sister group with *Epinephelus stictus*. More mitogenomic data from Epinephelidae species are essential to understand its taxonomic status with the family Serranidae.

## 1. Introduction

Groupers, a clade containing many commercial species, have long been classified as a subfamily to the family Serranidae (Perciformes) and common hinds including 234 species in 32 genera [1,2]. Craig et al. revised the classification of groupers based on genetic data [3,4]. The family Epinephelidae which includes 16 genera was officially proposed [5,6] and a growing stream of research prefers to accept this view [7,8,9]. However, the subfamily Epinephelinae is still used in a few recent studies [2,10]. This has caused great obstacles to the survey and conservation of grouper resources, and signifies that the classification status of Epinephelidae merits further investigation [3,11]. In addition, with the significant increase in catches and the aggravation of marine environmental pollution, the living conditions of groupers have been greatly threatened. Some grouper species (20 species to be exact) have even been listed as Critically Endangered (CR), endangered species (EN), or Vulnerable species (VU) by the International Union for Conservation of Nature (IUCN) [12].

Molecular biology has been applied to the studies on the classification and phylogeny of fishes and has solved some taxonomic problems [13]. Based on traditional morphology, the genus *Cephalopholis* should be classified as a subgenus of *Epinephelus*, however, many related studies have refuted this view and suggested that the genus should be an independent genus at both morphological and molecular levels [3,14]. The evolutionary biology of groupers has been widely studied by researchers, but the current understanding of the structure, evolution, characteristics, and properties of groupers’ mitogenomes is quite limited. To fill the gap in genetic information, this study analyzes the phylogeny and evolutionary histories of Epinephelidae based on the newly complete mitogenomes of four studied groupers with the published Epinephelidae data.

*Cephalopholis leopardus* (Lacepède, 1801) has a continuous dorsal fin with IX spines and 13 to 15 rays, and a pinkish-brown flat body with dense red-orange or pinkish red spots [15,16]. *Cephalopholis spiloparaea* (Valenciennes, 1828) is similar to the *C. leopardus* in appearance, except *C. spiloparaea* has 14 to 16 rays on the dorsal fin [15]. *Epinephelus amblycephalus* (Bleeker, 1857) and *Epinephelus hexagonatus* (Forster, 1801) are easier to distinguish in appearance. The *E. hexagonatus* is pale grey covered with hexagonal spots while *E. amblycephalus* has five broad dark brown bars; another difference is that the dorsal fin of *E. hexagonatus* and *E. amblycephalus* have 15 to 16 rays and 15 to 17 rays, respectively [4,5]. Genus *Cephalopholis* (Bloch and Schneider, 1801) was transitorily resurrected from the synonymy of *Epinephelus* species (Bloch, 1793) where it had lain dormant since its original description in 1801 [17]. As a valid genus, *Cephalopholis* was widely used and demoted to subgeneric status, but in subsequent publications it has again been recognized as the *Cephalopholis* genus [18,19]. Recognition of *Cephalopholis* as either a genus or subgenus is a demonstrated point, and *Cephalopholis* is a convenient taxon that is readily separable from other genera of groupers [20]. The genus *Cephalopholis* belongs to the family Epinephelidae, mainly distributed in the Indo Pacific region, with one dorsal fin with IX spines and 13 to 17 rays. The *C. spiloparaea* is similar to *C. leopardus* in shape and has IX spines, 14 to 16 rays, and 13 to 15 rays on its dorsal fin. Different from the genus *Cephalopholis*, *Epinephelus* species have a continuous dorsal fin with XI spines and 15 to 17 rays, which is oblong, flat, and stout, and is generally found near coral or rocky reefs in depths of 10 to 200 m around the Indo Pacific region [1].

Generally, the fish mitochondrial genome (mitogenome) is a circular, covalently closed double-stranded DNA molecule, which has maternal inheritance, conserved gene arrangement, and a high evolutionary rate [21,22]. Hence, it is considered to have an evolutionary origin independent of nuclear DNA. The length of the mitogenome is 15–20 kb, usually comprising 13 protein-coding genes (PCGs), 22 transfer RNA genes (tRNAs), 2 ribosomal RNA genes (rRNAs), 1 light-strand origin of replication (O_L_), and 1 control region (D-loop) [22]. There are some evolutionary studies focusing on Epinephelidae using mitogenome analysis [4], but the phylogenetic problems of Epinephelidae remain unclear because the evolutionary history is still not completely understood. In the present study, we first describe four complete mitogenomes of Epinephelidae (*Cephalopholis leopardus*, *Cephalopholis spiloparaea*, *Epinephelus amblycephalus*, and *Epinephelus hexagonatus*), providing potential markers for population genetic studies.

## 2. Materials and Methods

### 2.1. DNA Extraction, Amplification, and Sequencing

**Figure 1:**
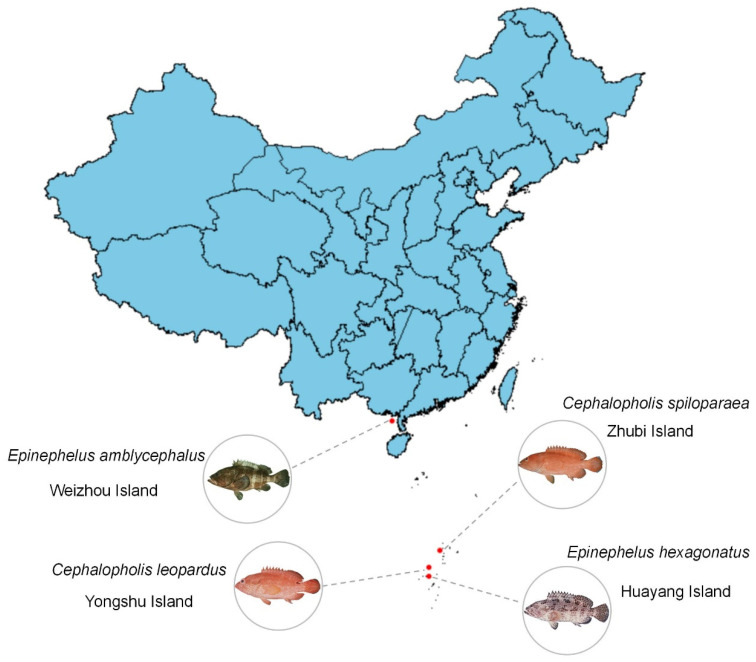
Sample collection sites in South China Sea, China.

Multiple specimens of four groupers were collected from four sites (Weizhou Dao, Huayang Jiao, Yongshu Jiao, and Zhubi Jiao) in the South China Sea, China (Figure 1, Table 1). The tissue samples of the four studied species were collected and immediately deposited in 95% alcohol, then stored at −20 °C. Genomic DNA was extracted from the gill using the Marine Animal Tissue Genomic DNA Extraction Kit (Qiagen, Beijing, China). The 2 × EasyTaq^®^ PCR SuperMix (Takara Biomedical Technology, Beijing, China) was used to amplify the segments using a long polymerase chain reaction (PCR) and primer walking method [23]. The parameters of the PCR reactions were followed the manufacturer’s recommendations to amplify DNA. The nine long fragments were separated from the complete mitogenomes of groupers with optimized universal primers of Epinephelidae mitogenome designed by Zhuang [24]. Then, the fragments of mitogenomes were obtained by sanger sequencing.

### 2.2. Sequence Assembly, Annotation, and Analysis

Sequence data were compiled to create four complete mitogenomes using the DNAStar v7.1 program [25]. Each species was identified by morphological identification and *COI* and *Cytb* genes were identified by Blast (https://blast.ncbi.nlm.nih.gov/Blast.cgi accessed on 12 December 2021). The four studied mitogenomes were first annotated with MITOS2 Web Server [26]. Then, tRNAs’ secondary structures were identified by online tRNAscan-SE Search Server v2.0 (Washington University School of Medicine, St Louis, MO, USA) [27], and further confirmed by ARWEN v1.2 online by default search mode [28]. Annotation and accurate boundary determination of PCGs and rRNAs genes were manually aligned and compared with the other released mitogenomes of Epinephelidae species using DNAman v6 software (Lynnon Biosoft, San Ramon, CA, USA) [29].

**Figure 2:**
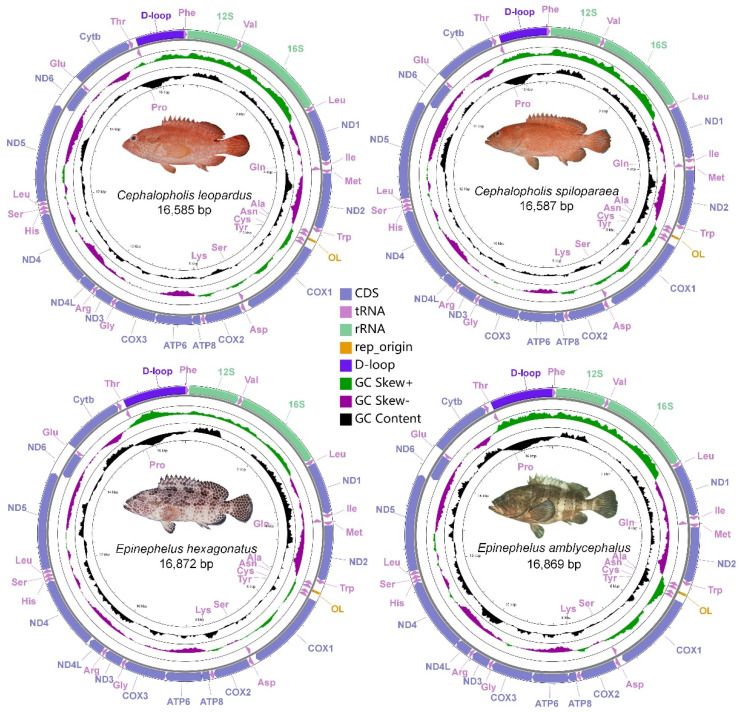
Graphical maps of the mitogenomes of *Cephalopholis leopardus*, *Cephalopholis spiloparaea*, *Epinephelus amblycephalus*, and *Epinephelus hexagonatus*.

The mitogenomes of the four groupers were visualized into complete circular genomes by the CGView Server v1.0 (Figure 2) [30]. The base compositions, codon usage, and relative synonymous codon usage (RSCU) values were calculated in the MEGA X program (Tokyo Metropolitan University, Tokyo, Japan) [31]. The map was drawn using ggblot2 by Rstudio [32]. Nucleotide diversity (Pi) of the PCGs in the four studied Epinephelidae mitogenomes were calculated with DnaSP v6 (Universitat de Barcelona, Barcelona, Spain) [33]. Non-synonymous substitution (Ka) and synonymous substitution (Ks) mutation rate ratios were performed by KaKs_Calculator 2.0 program [34]. The following formulas was used to measured strand asymmetry of the basis: AT skew = [(A − T)/(A + T)] and GC skew = [(G − C)/(G + C)] [35].

### 2.3. Phylogenetic Analysis

Phylogenetic analyses were conducted using the four newly sequenced mitogenomes and 43 Epinephelidae species available in the National Center of Biotechnology Information (NCBI) (https://www.ncbi.nlm.nih.gov/, accessed on 30 December 2021) (Table S1). *Lates japonicus* (NC_034339) and *Pagrus major* (NC_003196) were used as outgroups. All DNA sequences of 12 H-strand coded protein genes and two rRNAs were aligned by the MACSE v.2.03 (http://mbb.univ-montp2.fr/macse, accessed on 30 December 2021) and MAFFT program [36,37], respectively. The alignments of 12 PCGs (except the start and stop codon) were extracted from the first and the second codon. Then, ambiguously aligned fragments of rRNAs were removed using Gblocks 0.91b (accessed on 30 December 2021) [38]. A dataset was comprised by concatenating the above three segments by Phylosuite v1.2.2 (Bio-Transduction Lab, Wuhan, China) [39].

The ModelFinder lugin integrated into PhyloSuite was used to select the best-fit partition model with the greedy algorithm [40]. GTR + F + I + G4 was selected as the optimal model for the three partitions, according to the Akaike information (AICc) criterion [41]. Phylogenetic analyses were performed using the ML and BI analyses methods, using IQ-TREE v1.6.2 software (Medical University of Vienna, Vienna, Austria) under 10,000 ultrafast bootstraps with the approximate Bayes test and MrBayes 3.2.6 program (University of California, La Jolla, San Diego, CA, USA) under two parallel runs and 2,000,000 generations [42,43]. The phylogenetic trees dataset files were used to visualize and annotate the phylograms using the online iTOL v6 platform [44], embellished by Adobe Illustrator CS6 software (Adobe Systems Incorporated, San Jose, CA, USA).

## 3. Results and Discussion

### 3.1. Genome Organization and Base Composition

The lengths of *C. leopardus*, *C. spiloparaea*, *E. amblycephalus*, and *E. hexagonatus* mitogenomes were 16,585 bp, 16,587 bp, 16,869 bp, and 16,872 bp, respectively (Table 1), and within the range of the other 37 published mitogenomes of groupers from 16,389 bp (*Epinephelus latifasciatus*) to 17,227 bp (*Epinephelus bleekeri*) (Table S1). The gene content of the four species was similar to the most well-known mitogenomes of groupers, including 13 PCGs, 22 tRNAs, 2 rRNAs, 1 control region (CR), and the origin of L-strand replication (O_L_) (Figure 2, Table 2). Eight tRNA (tRNA-*Ala*, tRNA-*Cys*, tRNA-*Glu*, tRNA-*Asn*, tRNA-*Pro*, tRNA-*Gln*, tRNA-*Ser* (TGA), and tRNA-*Tyr*) and *ND6* genes were located on the light strand (L-strand), while other genes (12 PCGs, 14 tRNAs, 2 rRNAs, and 1 D-loop) were encoded on the heavy strand (H-strand). The size and location of each gene were similar in *Cephalopholis* and *Epinephelus*; the lengths of *Cephalopholis* mitogenomes presented only two bp differences, and only three bp differences in *Epinephelus*.

**Figure 3:**
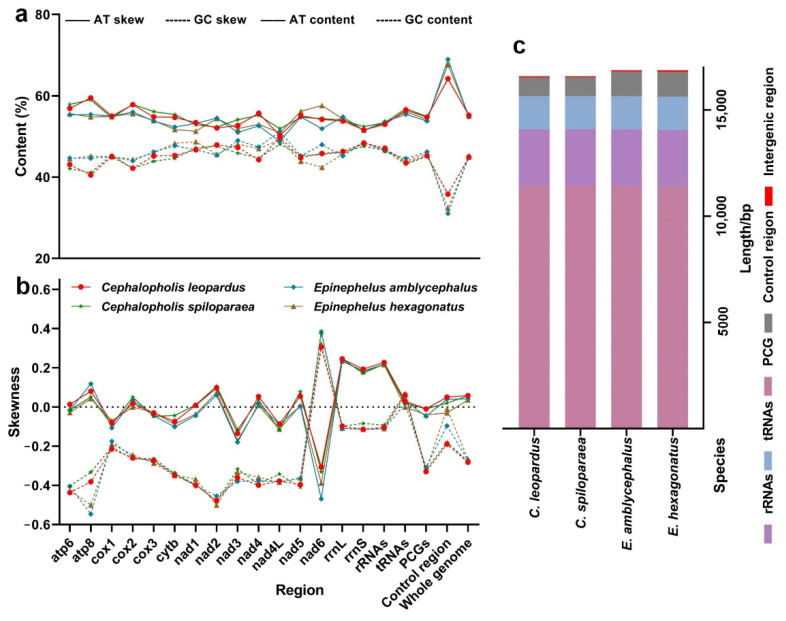
Base composition (**a**), skewness (**b**), and gene length (**c**) of the complete mitogenomes of the four groupers.

According to the base composition, the AT content of the *C. leopardus*, *C. spiloparaea*, *E. amblycephalus*, and *E. hexagonatus* mitogenomes were 55.2%, 55.3%, 54.8%, and 55.0%, respectively. There is less variation in nucleotide composition among the mitogenomes of the four groupers; all showed a higher AT content than GC content (Figure 3a). The AT-skews of the complete mitogenomes among four groupers are positive, while the GC-skews are all negative (Figure 3b). Except for the *ND6* gene, the AT-skews of 12 L-strand PCGs are more positive than GC-skews. These results show that the A content is higher than T, and the C content is higher than the G content for studied mitogenomes. The mitogenomes of the four species were found to be enriched in G and T bases in the H-strand and A and C bases in the L-strand. In the *ND6* gene, the GC-skew value is completely opposite to 12 H-strand PCGs, a phenomenon commonly found in vertebrates [45].

### 3.2. Protein Coding Genes and Codon Usage

Similar to other fish, PCGs of the mitogenomes of four groupers were encoded on the H-strand, except for the *ND6* gene that is located on the L-strand. The length of PCGs ranged from 11,406 bp (*E. hexagonatus*) to 11,430 bp (*C. leopardus* and *C. spiloparaea*). The majority of the PCGs were initiated with ATG or GTG (*COI* gene), but *ATP6* of the four groupers started with CTG, different from most other teleost and similar to the published mitogenomes of Epinephelidae (Table S1). All PCGs of the studied groupers use the typical TAA (includes the abbreviated T- form) stop codons. As the typical termination codon in many mitogenomes, TAG is not used frequently by the PCGs among the four groupers. The 12 PCGs encoded by the H-strand displayed T rich, while the *ND6* gene located in the L-strand displayed T rich (Figure 3a,b).

**Figure 4:**
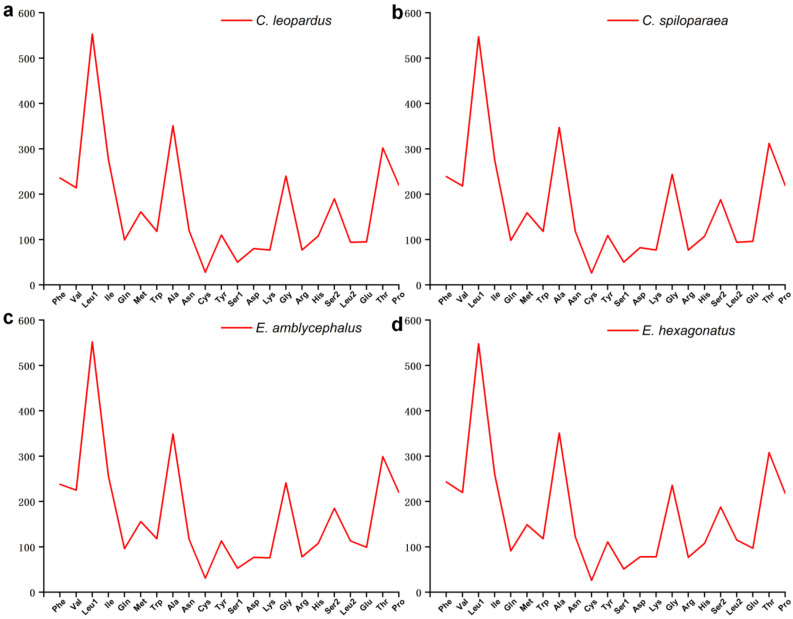
The amino acid usage of the complete mitogenomes of the four groupers (**a**–**d**).

**Figure 5:**
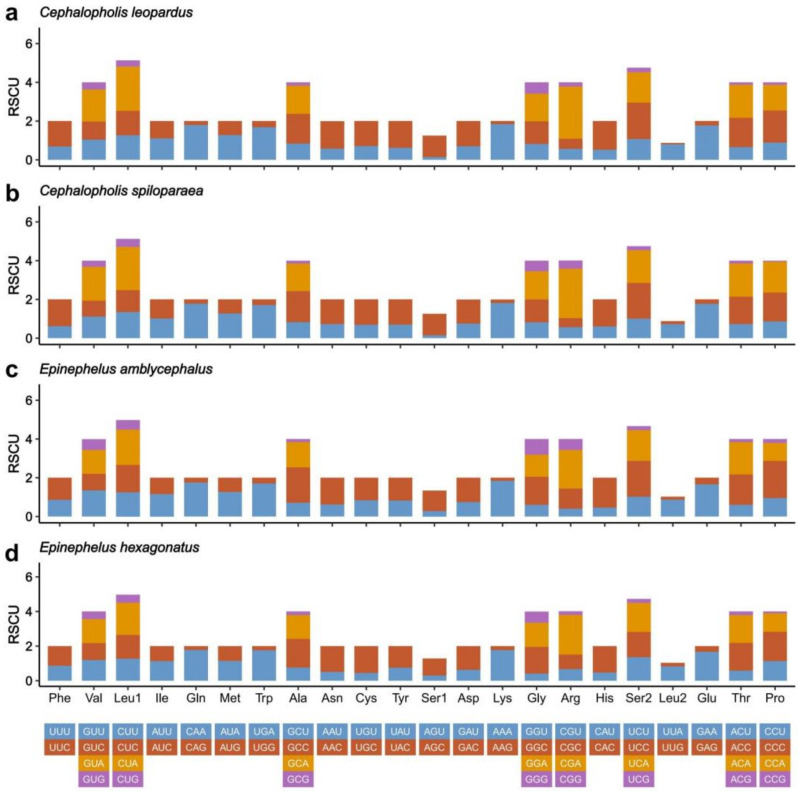
Relative synonymous codon usage (RSCU) in the mitogenomes of *Cephalopholis leopardus* (**a**), *Cephalopholis spiloparaea* (**b**), *Epinephelus amblycephalus* (**c**) and *Epinephelus hexagonatus* (**d**) mitogenomes.

The codon usage of PCGs is 3793 in *E. hexagonatus* and 3800 in the other three species. The frequent amino acids in the PCGs of the four studied mitogenomes were Leucine (Leu) and Alanine (Ala), visualized in Figure 4, similar to those being used in other Epinephelidae mitogenomes. In addition, the minimally used amino acid in the four groupers’ mitogenomes was Cysteine (Cys). At the same time, the relative synonymous codon usage (RSCU) values of four studied mitogenomes were calculated and visualized (Figure 5). The result showed that the third codons were biased to use A and T rather than C and G in the four groupers.

In the thirteen PCGs of the four studied mitogenomes, Leu (CUU, CUC, CUA, and CUG) and Ala (GCU, GCC, GCA, and GCG) were the most abundant amino acids, and Cys (UGU and UGC) and Arg (CGU, CGC, CGA, and CGG) were rarely used. The RSCU is usually used to assess the synonymous codon usage bias. The RSCU showed that the codons end in A or T, more than G or C, similar to other fish mitogenomes [46]. Generally, codon usage was related to the gene expression level, the function of encoded proteins, tRNA abundance, and base composition [47,48,49,50,51]. Overall, the length, RSCU, skewness, and AT content of 13 PCGs in the Genus *Cephalopholis* and *Epinephelus* mitogenomes were nearly identical (Figure 3, Figure 4 and Figure 5). This means that the four studied groupers might be related to natural selection.

### 3.3. Sliding Window Analysis and Nucleotide Diversity

**Figure 6:**
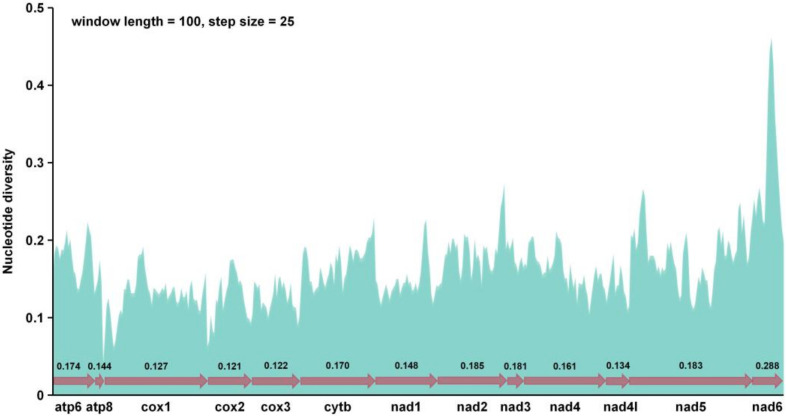
Sliding window analysis based on thirteen PCGs among four groupers. The red line shows the value of nucleotide diversity in the sliding window analysis window size = 100 bp, step size = 25 bp. The length of the arrow represents the length of 13 PCGs.

To reveal the evolutionary pattern of the PCGs, the Ka/Ks and nucleotide diversity of the four studied mitogenomes were calculated for the aligned thirteen PCGs. Nucleotide diversity was analyzed with a sliding window of the four studied groupers (Figure 6). The plot of sequence variation ratio exhibited highly variable nucleotide diversity among the four sequences, with nucleotide diversity values for the 100 bp windows ranging from 0.038 to 0.387. The nucleotide diversity values of most genes were similar. The *COII* gene had the lowest nucleotide diversity of 0.121, and the *ND6* gene had significantly higher nucleotide diversity (0.288) than other genes.

**Figure 7:**
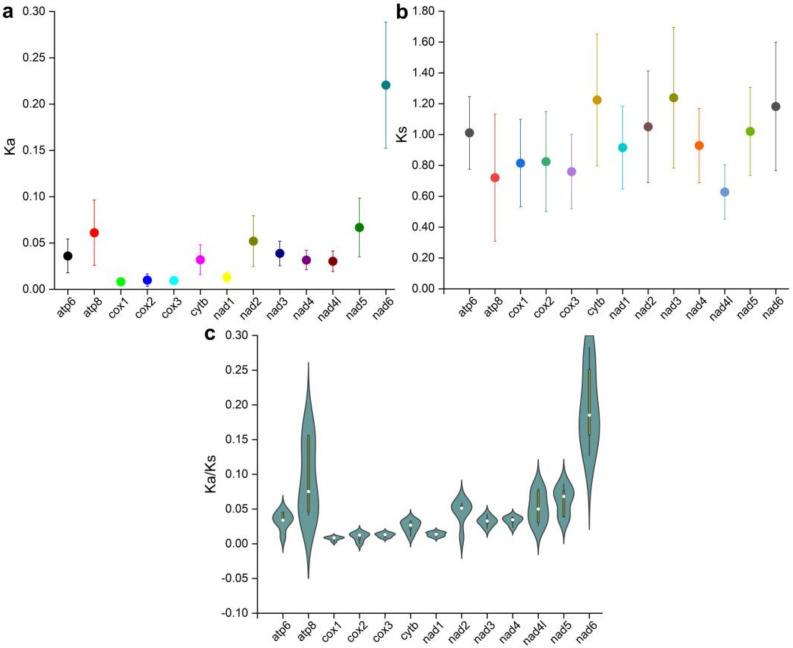
The average non-synonymous (Ka) substitution rates (**a**), synonymous (Ks) substitution rates (**b**) and Ka/Ks (**c**) of 13 coding proteins among four species.

As a universal indicator, Ka/Ks, the ratio of nonsynonymous substitution (Ka) and synonymous substitution (Ks), is usually used to assess selective pressure, even evolutionary relationships between different species in molecular studies [52,53,54]. Generally, Ka/Ks < 1, Ka/Ks = 1, and Ka/Ks > 1 were represented by the purifying selection, neutral mutation, and positive selection, respectively [55]. Ka/Ks ratios were estimated to investigate the evolutionary rates of all 13 PCG genes. The Ka/Ks ratio of all PCGs was less than 0.30, which indicated that the four sequences had a high similarity. The *ATP8* and *ND6* genes exhibited relaxed purifying selection, while other PCGs were under the strongest purifying selection (Figure 7). The ratio of Ka and Ks is the representative parameter of selective pressure magnitude and direction [56]. It indicated that four groupers were suffering a purifying selection. Among the 13 PCG genes, the average Ka/Ks ratio is higher in *ND6* and *ATP8* genes, implying they are under less purifying selection, similar to the mitogenomes of vertebrates and mollusks [57].

### 3.4. Transfer RNA and Ribosomal RNA Genes

**Figure 8:**
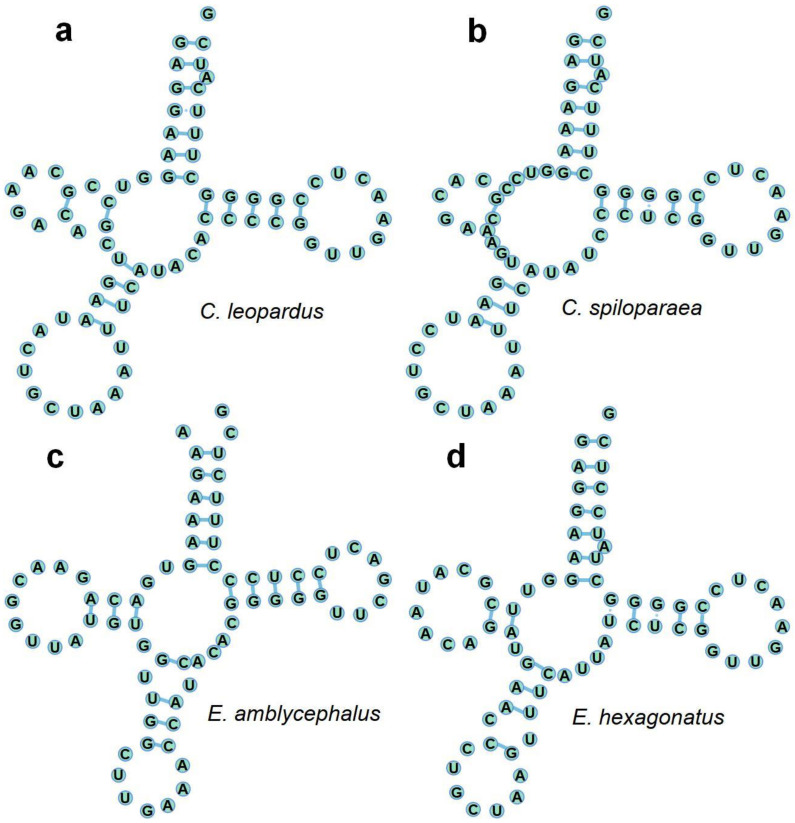
The tRNA-*Ser*(AGY) in the mitogenomes of *Cephalopholis leopardus* (**a**), *Cephalopholis spiloparaea* (**b**), *Epinephelus amblycephalus* (**c**) and *Epinephelus hexagonatus* (**d**).

Similar to the typical set of tRNA genes in Epinephelidae mitogenomes, all 22 standard tRNAs were found with high AT content (55.4–56.9%). However, the DHU stem was extremely conserved [7]. The length of tRNAs was similar to the four grouper mitogenomes, ranging from 1561 bp (*E. amblycephalus*) to 1565 bp (*C. spiloparaea*), and AT content was higher than GC content (Figure 3a,c). All tRNA (all four groupers compared simultaneously) varied in length from 67 bp (tRNA-*Cys*) to 76 bp (tRNA-*Leu*). There were two tRNA-Leu and tRNA-Ser among the twenty-two predicted tRNA genes from the four groupers (Table 2). All the predicted tRNAs displayed the typical cloverleaf secondary structure, except for tRNA-*Ser* (AGY), which cannot form a stable secondary structure because of the lack of the DHU arm; this structure was common among grouper mitogenomes [7]. The secondary cloverleaf structures of tRNA-*Ser* (AGY) genes identified in the mitogenome of the four groupers are shown in Figure 8.

The mitogenomes of the four groupers had one 12S rRNA and one 16S rRNA gene, located between tRNA-*Phe* and tRNA-*Val*, and between tRNA-*Val* and tRNA-*Leu* (UUR), respectively. Among four groupers, the size of 12S rRNAs ranged from 952 bp (*E. amblycephalus*) to 956 bp (*E. hexagonatus*), and 16S rRNAs ranged from 1705 bp (*C. spiloparaea*) to 1710 bp (*E. amblycephalus* and *E. hexagonatus*) in length. The two rRNAs were both encoded by the H-strand, a moderate nucleotide compositional with more AT content (53.0–53.6%) than GC content (46.4–47.1%), which is shown in Figure 3a.

### 3.5. Non-Coding Region

The non-coding regions in the mitogenomes of the four groupers include the CR, O_L_, and several short intergenic spacers. The intergenic spacers included the non-coding (NC) sequence and overlap region. The NCs ranged from 1 to 20 bp in size. The CR is the largest of these non-coding regions in which the groupers’ mitogenomes were located between tRNA-*Pro* and tRNA-*Phe* in the mitogenome of groupers, ranging from 880 bp (*C. leopardus* and *C. spiloparaea*) to 1172 bp in *E. hexagonatus*. The base composition of CR with AT rich ranged from 64.1% (*C. spiloparaea*) to 69.0% (*E. amblycephalus*). The length of the CR is variable, which is the main reason for the differences in the mitogenome lengths in fishes. The CR of all studied species’ mitogenomes showed positive AT-skew values while all control regions of the four grouper species displayed negative GC-skew values.

The O_L_ of the four studied mitogenomes commonly lies between tRNA-*Asn* and tRNA-*Cys* with the length ranging from 34 bp (*C. spiloparaea*) to 44 bp (*E. amblycephalus*). Four of the studied groupers shared common features of the vertebrate O_L_, a pyrimidine rich 5′ flanking region of the stem (Table 3), similar to the published Epinephelidae mitogenomes. In addition, the length of nine overlap regions in two *Cephalopholis* ranged from one to 10 bp, and the genus *Epinephelus* had seven overlap regions from one to 16 bp in size.

### 3.6. Phylogenetic Analysis

**Figure 9:**
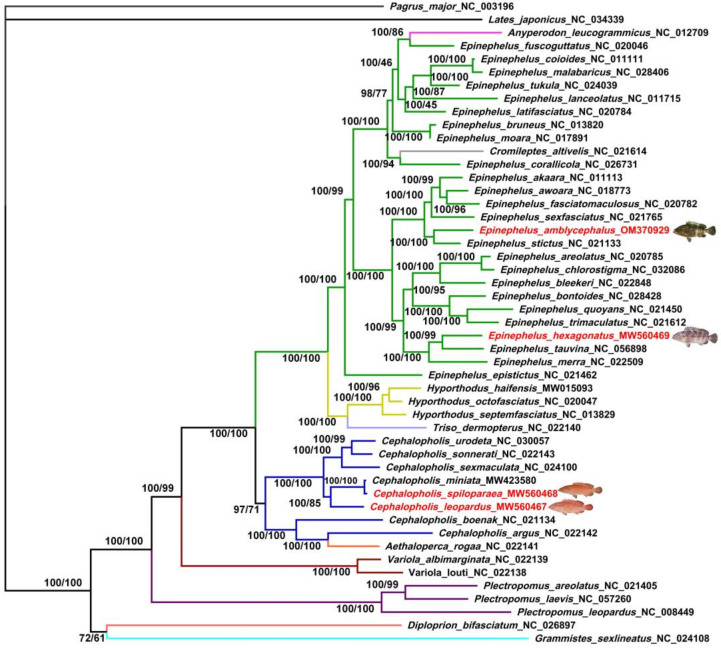
The phylogenetic tree inferred from the nucleotide sequences of twelve H-strand PCGs (the first and second codons) and two rRNAs using the Bayesian inference (BI) and maximum likelihood (ML) methods. Numbers on branches indicate posterior probability (**left**) and bootstrap (**right**).

To further investigate the phylogenetic relationships of the family Epinephelidae, phylogenetic trees were conducted with the concatenated mitogenomic sequences of 47 groupers and 2 outgroups. Phylogenetic analyses based on both Maximum Likelihood (ML) and Bayesian analyses (BI) methods revealed identical topologies, which also generally agreed with those presented in previous studies. The high nodal support value topologies of the two methods were shown by high bootstraps (left) and posterior probabilities (right) values (Figure 9). Contrary to the traditional classification, the molecular phylogenetic tree showed three clades of *Epinephelus* species, and the genera *Cephalopholis* and *Epinephelus* are not monophyletic groups. Genera *Anyperodon* and *Cromileptes* clustered to *Epinephelus* species as sister groups rather than only genus *Epinephelus. Epinephelus hexagonatus* is close to *E. tauvina* and *E. merra*, and *E. amblycephalus* is a sister group of *E. stictus*. *Cephalopholis* *leopardus*, *C. spiloparaea*, and *C. miniata* are located in a clade far away from *Epinephelus* species. Except for *Aethaloperca rogaa* and *C. argus* assembling a clad, the other *Cephalopholis* species are assumed to be a monophyletic group and close to the genus *Variola*. In addition, *Triso dermopterus* is a sister group to the *Hyporthodus* species, and close to the genera *Cephalopholis* and *Epinephelus.*

To test classifications on the mitogenome level, identical topologies of the two methods were produced by high nodal support values. The results showed that the genus *Cephalopholis* and *Epinephelus* are not monophyletic groups; this result was also seen by the non-monophyly of two grouper genera using the mitochondrial 16S rRNA gene by Craig [3]. The phylogenetic tree showed that the location of the two grouper genera was far, and *Cephalopholis* species should be elevated from the subgenus *Cephalopholis* to the genus *Cephalopholis* [1]. The taxonomic status of the *Cephalopholis* and *Epinephelus* genus is well supported by the results of a phylogenetic tree, consistent with previous studies [7,58,59]. In addition, the genera *Aethaloperca*, *Anyperodon*, *Cromileptes*, *Cephalopholis*, *Epinephelus*, *Hyporthodus*, *Triso*, *Plectropomus*, and *Variola* clustered with a strong monophyletic branch, which is consistent with the new classification of Epinephelidae [5,6]. The present study will greatly improve our understanding of the evolution profile and phylogenetic position in the family Epinephelidae.

## 4. Conclusions

Epinephelidae is a large family including 9 genera with at least 160 recognized species [5,6]. There are only 43 published whole mitogenome sequences of Epinephelidae in NCBI, 2022. The complete mitogenomes of four groupers which were first identified and compared in this study could increase the essential molecular data of Epinephelidae. The length of the mitogenomes of *C. leopardus*, *C. spiloparaea*, *E. amblycephalus*, and *E. hexagonatus* ranged from 16,585 bp, 16,587 bp, 16,869 bp, and 16,872 bp, respectively. The genomic structure of four groupers’ mitogenomes is conserved and similar to vertebrates, with typical gene order and few differences. Moreover, the AT-skew of the rRNAs was strongly positive whereas the GC-skew was slightly negative, showing that the contents of A and C were higher than those of T and G in the rRNAs among four groupers, respectively.

Due to growth characteristics in groupers, only morphological identification is prone to error. The mitogenome has been used as an effective tool for phylogenetic and population genetic analyses in fish. The insufficient number of species could cause questionable phylogenetic results; more grouper species will be needed in molecular studies for further investigation [60,61].

## Figures and Tables

**Table 1:** Detailed information on the four analyzed Epinephelidae species.

Species	GenBank Accession Number	Size (bp)	AT%	AT-Skew	Sampling ID	Sampling Year
*Cephalopholis leopardus*	MW560467	16,585	55.2	0.06	YS2018052107	2018
*Cephalopholis spiloparaea*	MW560468	16,587	55.3	0.06	ZB2018050814	2018
*Epinephelus amblycephalus*	OM370929	16,872	55.0	0.04	WZ2018110410	2019
*Epinephelus hexagonatus*	MW560469	16,869	54.8	0.04	HY2019060508	2019

**Table 2:** Features of the complete mitogenomes of four Epinephelidae.

Genes	Strand	Size (bp)	Start Codon	Stop Codon	Anticodon
*Cephalopholis leopardus/Cephalopholis spiloparaea/Epinephelus amblycephalus/Epinephelus hexagonatus*					
tRNA-*Phe* (F)	H	70/69/70/70			GAA
12S rRNA	H	954/954/952/956			
tRNA-*Val* (V)	H	71/71/70/71			TAC
16S rRNA	H	1709/1705/1710/1710			
tRNA-*Leu*1 (L1)	H	75/76/76/75			TAA
*ND1*	H	975/975/975/975	ATG/ATG/ATG/ATG	TAA/TAA/TAA/TAA	
tRNA-*Ile* (I)	H	70/70/70/70			TTG
tRNA-*Gln* (Q)	L	71/71/71/71			GAT
tRNA-*Met* (M)	H	69/69/69/69			CAT
*ND2*	H	1047/1047/1047/1047	ATG/ATG/ATG/ATG	TAA/TAA/TAA/TAA	
tRNA-*Trp* *(W)*	H	71/71/71/71			TCA
tRNA-*Ala* (A)	L	69/69/69/69			TGC
tRNA-*Asn* (N)	L	73/73/73/73			GTT
O_L_	-	41/40/44/43			
tRNA-*Cys* (C)	L	68/67/67/68			GCA
tRNA-*Tyr* (Y)	L	71/71/71/71			GTA
*COI*	H	1551/1551/1551/1551	GTG/GTG/GTG/GTG	TAG/TAA/TAA/TAG	
tRNA-*Ser*1 (S1)	L	72/71/71/71			TGA
tRNA-*Asp* (D)	H	73/74/73/73			GTC
*COII*	H	691/691/691/691	ATG/ATG/ATG/ATG	T-/T-/T-/T-	
tRNA-*Lys* (K)	H	73/73/74/73			TTT
*ATP8*	H	168/168/168/168	ATG/ATG/ATG/ATG	TAA/TAA/TAA/TAA	
*ATP6*	H	684/690/684/684	CTG/CTG/CTG/CTG	TAA/TAA/TAA/TAA	
*COIII*	H	786/786/786/786	ATG/ATG/ATG/ATG	TAA/TAA/TAA/TAA	
tRNA-*Gly* (G)	H	72/72/72/72			TCC
*ND3*	H	351/349/349/351	ATG/ATG/ATG/ATG	TAG/T-/T-/TAG	
tRNA-*Arg* (R)	H	69/69/69/69			TCG
*ND4L*	H	297/297/297/297	ATG/ATG/ATG/ATG	TAA/TAA/TAA/TAA	
*ND4*	H	1381/1354/1381/1381	ATG/ATG/GTG/ATG	T-/T-/T-/T-	
tRNA-*His* (H)	H	71/70/70/70			GTG
tRNA-*Ser*2 (S2)	H	72/71/70/72			GCT
tRNA-*Leu*2 (L2)	H	73/73/73/73			TAG
*ND5*	H	1839/1839/1839/1839	ATG/ATG/ATG/ATG	TAA/TAA/TAA/TAA	
*ND6*	L	522/522/522/522	ATG/ATG/ATG/ATG	TAA/TAG/TAA/TAG	
tRNA-*Glu* (E)	L	69/69/69/69			TTC
*Cytb*	H	1141/1141/1141/1141	ATG/ATG/ATG/ATG	T-/T-/T-/T-	
tRNA-*Thr* (T)	H	73/73/73/73			TGT
tRNA-*Pro* (P)	L	70/70/70/70			TGG
Control region (CR)	H	880/880/1170/1171			

**Table 3:** Detailed information of O_L_ in four studied mitogenomes.

Species	Length (bp)	Sequence
*Cephalopholis leopardus*	35	TCCCCCGCCTAATAATAAGACTAATAGGCGGGGGA
*Cephalopholis spiloparaea*	34	TCCCCCGCCTAATAATAGGCTAATAGGCGGGGGA
*Epinephelus amblycephalus*	44	GCTTCCCCCCGCCCCCCGGGGGGCGGGGGGCGGCGGGGGGAAGC
*Epinephelus hexagonatus*	39	TCCCCCGCCTACTCAGATCCAAAAGGAGTAGGCGGGGGA

## Data Availability

Not applicable.

## Supplementary Materials

The following supporting information can be downloaded at: https://www.mdpi.com/article/10.3390/genes13040660/s1, Table S1: Detailed information on the analyzed species in this study.

## References

[1] Heemstra P.C., Randall J.E. (1993). FAO Species Catalogue. Groupers of the World (Family Serranidae, Subfamily Epinephelinae). An Annotated and Illustrated Catalogue of the Grouper, Rockcod, Hind, Coral Grouper and Lyretail Species Known to Date. FAO Fish. Synop..

[2] Nelson J.S., Grande T.C., Wilson M.V. (2016). Fishes of the World.

[3] Craig M.T., Pondella D.J., Franck J.P., Hafner J.C. (2001). On the status of the Serranid fish genus *Epinephelus*: Evidence for paraphyly based upon 16S rDNA sequence. Mol. Phylogenet. Evol..

[4] Craig M.T., Hastings P.A. (2007). A molecular phylogeny of the groupers of the subfamily Epinephelinae (Serranidae) with a revised classification of the Epinephelini. Ichthyol. Res..

[5] Craig M.T., de Mitcheson Y.S., Heemstra P.C. (2011). Groupers of the World: A Field Market Guide.

[6] Ma K.Y., Craig M.T. (2018). An Inconvenient Monophyly: An Update on the Taxonomy of the Groupers (Epinephelidae). Copeia.

[7] Zhuang X., Qu M., Zhang X., Ding S. (2013). A comprehensive description and evolutionary analysis of 22 grouper (perciformes, epinephelidae) mitochondrial genomes with emphasis on two novel genome organizations. PLoS ONE.

[8] Luiz O.J., Woods R.M., Madin E.M., Madin J.S. (2016). Predicting IUCN extinction risk categories for the world’s data deficient groupers (Teleostei: Epinephelidae). Conserv. Lett..

[9] Vaini J.O., Mota K.G., Ojeda A.P., Barreiros J.P., Moreira R.G., Hilsdorf A.W. (2019). Development and characterization of 20 polymorphic microsatellite markers for *Epinephelus marginatus* (Lowe, 1834) (Perciformes: Epinephelidae) using 454 pyrosequencing. Genet. Mol. Biol..

[10] Betancur R.R., Wiley E.O., Arratia G., Acero A., Bailly N., Miya M., Lecointre G., Orti G. (2017). Phylogenetic classification of bony fishes. BMC Evol. Biol..

[11] Tucker S.J., Kurniasih E.M., Craig M.T. (2016). A New Species of Grouper (*Epinephelus*; Epinephelidae) from the Indo-Pacific. Copeia.

[12] De Mitcheson Y.S., Craig M.T., Bertoncini A.A., Carpenter K.E., Cheung W.W.L., Choat J.H., Cornish A.S., Fennessy S.T., Ferreira B.P., Heemstra P.C. (2013). Fishing groupers towards extinction: A global assessment of threats and extinction risks in a billion dollar fishery. Fish Fish..

[13] Liu Z.J., Cordes J.F. (2004). DNA marker technologies and their applications in aquaculture genetics. Aquaculture.

[14] Baldwin C.C., Johnson G.D. (1993). Phylogeny of the epinephelinae (Teleostei: Serranidae). Bull. Mar. Sci..

[15] Myers R.F. (1999). Micronesian Reef Fishes: A Comprehensive Guide to the Coral Reef Fishes of Micronesia.

[16] Allen G.R., Erdmann M.V. (2012). Reef Fishes of the East Indies.

[17] Jordan D.S., Evermann B.W. (1905). The Aquatic Resources of the Hawaiian Islands: The Shore Fishes of the Hawaiian Islands, with a General Account of the Fish Fauna.

[18] Smith C.L. (1971). A revision of the American groupers: *Epinephelus* and allied genera. Bull. Am. Mus. Nat. Hist..

[19] Smith C.L., Fischer W., Bianchi G., Scott W.B. (1981). Family Serranidae. FAO Species Identification Sheets for Fishery Purposes, Eastern Central Altantic.

[20] Smith-Vaniz W.F., Johnson G.D., Randall J.E. (1988). Redescription of *Gracila albomarginata* (Fowler and Bean) and *Cephalopholis polleni* (Bleeker) with comments on the generic limits of selected Indo-Pacific groupers (Pisces: Serranidae: Epinephelinae). Proc. Acad. Nat. Sci. Phila..

[21] Wolstenholme D.R. (1992). Animal Mitochondrial DNA: Structure and Evolution. Int. Rev. Cytol..

[22] Boore J.L. (1999). Animal mitochondrial genomes. Nucleic Acids Res..

[23] Miya M., Nishida M. (1999). Organization of the Mitochondrial Genome of a Deep-Sea Fish, *Gonostoma gracile* (Teleostei: Stomiiformes): First Example of Transfer RNA Gene Rearrangements in Bony Fishes. Mar. Biotechnol..

[24] Zhuang X., Ding S.X., Wang J., Wang Y., Su Y. (2009). A set of 16 consensus primer pairs amplifying the complete mitochondrial genomes of orange-spotted grouper (*Epinephelus coioides*) and Hong Kong grouper (*Epinephelus akaara*). Mol. Ecol. Resour..

[25] Burland T.G. (2000). DNASTAR’s Lasergene sequence analysis software. Methods Mol. Biol..

[26] Bernt M., Donath A., Juhling F., Externbrink F., Florentz C., Fritzsch G., Putz J., Middendorf M., Stadler P.F. (2013). MITOS: Improved de novo metazoan mitochondrial genome annotation. Mol. Phylogenet. Evol..

[27] Lowe T.M., Chan P.P. (2016). tRNAscan-SE On-line: Integrating search and context for analysis of transfer RNA genes. Nucleic Acids Res..

[28] Laslett D., Canback B. (2008). ARWEN: A program to detect tRNA genes in metazoan mitochondrial nucleotide sequences. Bioinformatics.

[29] Wang W. The Molecular Detection of Corynespora Cassiicola on Cucumber by PCR Assay Using DNAman Software and NCBI. Proceedings of the Computer and Computing Technologies in Agriculture IX.

[30] Grant J.R., Stothard P. (2008). The CGView Server: A comparative genomics tool for circular genomes. Nucleic Acids Res..

[31] Kumar S., Stecher G., Li M., Knyaz C., Tamura K. (2018). MEGA X: Molecular Evolutionary Genetics Analysis across Computing Platforms. Mol. Biol. Evol..

[32] Wickham H. (2016). Ggplot2: Elegant Graphics for Data Analysis.

[33] Rozas J., Ferrer-Mata A., Sanchez-DelBarrio J.C., Guirao-Rico S., Librado P., Ramos-Onsins S.E., Sanchez-Gracia A. (2017). DnaSP 6: DNA Sequence Polymorphism Analysis of Large Data Sets. Mol. Biol. Evol..

[34] Wang D., Zhang Y., Zhang Z., Zhu J., Yu J. (2010). KaKs_Calculator 2.0: A Toolkit Incorporating γ-Series Methods and Sliding Window Strategies. Genom. Proteom. Bioinform..

[35] Katoh K., Standley D.M. (1995). Patterns of nucleotide composition at fourfold degenerate sites of animal mitochondrial genomes. J. Mol. Evol..

[36] Ranwez V., Douzery E.J.P., Cambon C., Chantret N., Delsuc F. (2018). MACSE v2: Toolkit for the Alignment of Coding Sequences Accounting for Frameshifts and Stop Codons. Mol. Biol. Evol..

[37] Katoh K., Standley D.M. (2013). MAFFT multiple sequence alignment software version 7: Improvements in performance and usability. Mol. Biol. Evol..

[38] Talavera G., Castresana J. (2007). Improvement of phylogenies after removing divergent and ambiguously aligned blocks from protein sequence alignments. Syst. Biol..

[39] Zhang D., Gao F., Jakovlić I., Zou H., Zhang J., Li W.X., Wang G.T. (2020). PhyloSuite: An integrated and scalable desktop platform for streamlined molecular sequence data management and evolutionary phylogenetics studies. Mol. Ecol. Resour..

[40] Kalyaanamoorthy S., Minh B.Q., Wong T.K.F., von Haeseler A., Jermiin L.S. (2017). ModelFinder: Fast model selection for accurate phylogenetic estimates. Nat. Methods.

[41] Burnham K.P., Anderson D.R. (2002). Model Selection and Multimodel Inference: A Practical Information-Theoretic Approach.

[42] Minh B.Q., Schmidt H.A., Chernomor O., Schrempf D., Woodhams M.D., von Haeseler A., Lanfear R. (2020). IQ-TREE 2: New Models and Efficient Methods for Phylogenetic Inference in the Genomic Era. Mol. Biol. Evol..

[43] Nylander J.A., Ronquist F., Huelsenbeck J.P., Nieves-Aldrey J.L. (2004). Bayesian phylogenetic analysis of combined data. Syst. Biol..

[44] Letunic I., Bork P. (2016). Interactive tree of life (iTOL) v3: An online tool for the display and annotation of phylogenetic and other trees. Nucleic Acids Res..

[45] Anderson S., Bankier A.T., Barrell B.G., de Bruijn M.H., Coulson A.R., Drouin J., Eperon I.C., Nierlich D.P., Roe B.A., Sanger F. (1981). Sequence and organization of the human mitochondrial genome. Nature.

[46] Wang C., Lai T., Ye P., Yan Y., Feutry P., He B., Huang Z., Zhu T., Wang J., Chen X. (2022). Novel duplication remnant in the first complete mitogenome of *Hemitriakis japanica* and the unique phylogenetic position of family Triakidae. Gene.

[47] Li B., Wang H., Yang L., Liu S., Zhuang Z. (2021). Complete Mitochondrial Genome of *Pseudocaranx dentex* (Carangidae, Perciformes) Provides Insight into Phylogenetic and Evolutionary Relationship among Carangidae Family. Genes.

[48] Gupta S.K., Majumdar S., Bhattacharya T.K., Ghosh T.C. (2000). Studies on the relationships between the synonymous codon usage and protein secondary structural units. Biochem. Biophys. Res. Commun..

[49] Moriyama E.N., Powell J.R. (1998). Gene length and codon usage bias in *Drosophila melanogaster*, *Saccharomyces cerevisiae* and *Escherichia coli*. Nucleic Acids Res..

[50] Moriyama E.N., Powell J.R. (1997). Codon usage bias and tRNA abundance in *Drosophila*. J. Mol. Evol..

[51] Carlini D.B., Chen Y., Stephan W. (2001). The relationship between third-codon position nucleotide content, codon bias, mRNA secondary structure and gene expression in the drosophilid alcohol dehydrogenase genes Adh and Adhr. Genetics.

[52] Boore J.L., Medina M., Rosenberg L.A. (2004). Complete sequences of the highly rearranged molluscan mitochondrial genomes of the Scaphopod *Graptacme eborea* and the bivalve *Mytilus edulis*. Mol. Biol. Evol..

[53] Dellaporta S.L., Xu A., Sagasser S., Jakob W., Moreno M.A., Buss L.W., Schierwater B. (2006). Mitochondrial genome of Trichoplax adhaerens supports placozoa as the basal lower metazoan phylum. Proc. Natl. Acad. Sci. USA.

[54] Zhang D., Zou H., Wu S.G., Li M., Jakovlic I., Zhang J., Chen R., Li W.X., Wang G.T. (2018). Three new Diplozoidae mitogenomes expose unusual compositional biases within the Monogenea class: Implications for phylogenetic studies. BMC Evol. Biol..

[55] Yang Z., Bielawski J.P. (2000). Statistical methods for detecting molecular adaptation. Trends Ecol. Evol..

[56] Stenico M., Lloyd A.T., Sharp P. (1994). Codon usage in *Caenorhabditis elegans*: Delineation of translational selection and mutational biases. Nucleic Acids Res..

[57] Monnens M., Thijs S., Briscoe A.G., Clark M., Frost E.J., Littlewood D.T.J., Sewell M., Smeets K., Artois T., Vanhove M.P.M. (2020). The first mitochondrial genomes of endosymbiotic rhabdocoels illustrate evolutionary relaxation of *atp8* and genome plasticity in flatworms. Int. J. Biol. Macromol..

[58] Liu M., Li J.L., Ding S.X., Liu Z.Q. (2013). *Epinephelus moara*: A valid species of the family Epinephelidae (Pisces: Perciformes). J. Fish Biol..

[59] Chen X., Chen H., Yang W., Lin L., Liu M. (2016). Complete mitochondrial genome and the phylogenetic position of the brown-spotted grouper *Epinephelus chlorostigma* (Perciformes: Epinephelidae). Mitochondrial DNA A DNA Mapp. Seq. Anal..

[60] Wang C., Chen H., Tian S., Yang C., Chen X. (2020). Novel Gene Rearrangement and the Complete Mitochondrial Genome of *Cynoglossus monopus*: Insights into the Envolution of the Family Cynoglossidae (Pleuronectiformes). Int. J. Mol. Sci..

[61] Satoh T.P., Miya M., Mabuchi K., Nishida M. (2016). Structure and variation of the mitochondrial genome of fishes. BMC Genom..

