# Supplementary material for: Comparative Analysis of Four Complete Mitochondrial Genomes of Epinephelidae (Perciformes)
Source: Genes (Basel). 2022 Apr 8;13(4):660. doi: 10.3390/genes13040660 (PMC9029768; doi:10.3390/genes13040660)
Supplement: Supplementary file 1 [file genes-13-00660-s001.zip › genes-1640524-supplementary.pdf]

Supplementary material

Table S1. Detailed information of the analyzed species in this study.

Species	Accession number	Size (bp)	AT%	Authors
Aethaloperca rogaa	NC_022141	16,538	56.7	Zhuang, *et al.*
Anyperodon leucogrammicus	NC_012709	16,616	55.6	Lin, *et al.*
Cephalopholis argus	NC_022142	16,767	56.9	Zhuang, *et al.*
Cephalopholis boenak	NC_021134	16,771	57.0	Li, *et al.*
Cephalopholis leopardus	MW560467	16,585	55.2	Chen, *et al.*
Cephalopholis miniata	MW423580	16,585	55.3	Gong, *et al.*
Cephalopholis sexmaculata	NC_024100	16,589	55.4	Hsiao, *et al.*
Cephalopholis sonnerati	NC_022143	16,587	55.8	Zhuang, *et al.*
Cephalopholis spiloparaea	MW560468	16,587	55.3	Chen, *et al.*
Cephalopholis urodeta	NC_030057	16,592	55.7	Guo, *et al.*
Cromileptes altivelis	NC_021614	16,497	55.3	Qin, *et al.*
Diploprion bifasciatum	NC_026897	16,805	53.9	Wang, *et al.*
Epinephelus akaara	NC_011113	16,795	56.0	Zhuang, *et al.*
Epinephelus amblycephalus	OM370929	16,869	54.8	Chen, *et al.*
Epinephelus areolatus	NC_020785	16,893	55.6	He, *et al.*
Epinephelus awoara	NC_018773	16,802	55.8	Qu, *et al.*
Epinephelus bleekeri	NC_022848	17,227	54.9	Shu, *et al.*
Epinephelus bontoides	NC_028428	16,903	55.9	Hsiao, *et al.*
Epinephelus bruneus	NC_013820	16,686	55.0	Oh, *et al.*
Epinephelus chlorostigma	NC_032086	16,894	55.7	Chen, *et al.*
Epinephelus coioides	NC_011111	16,418	55.2	Zhuang, *et al.*
Epinephelus corallicola	NC_026731	16,647	55.3	Zheng, *et al.*
Epinephelus epistictus	NC_021462	16,920	55.4	Peng, *et al.*
Epinephelus fasciatomaculosus	NC_020782	16,682	55.8	Li, *et al.*
Epinephelus fuscoguttatus	NC_020046	16,648	56.1	Zhuang, *et al.*
Epinephelus hexagonatus	MW560469	16,872	55.0	Chen, *et al.*
Epinephelus lanceolatus	NC_011715	16,574	55.9	Chou, *et al.*
Epinephelus latifasciatus	NC_020784	16,389	55.0	Lai, *et al.*
Epinephelus malabaricus	NC_028406	16,423	55.3	Zhu, *et al.*
Epinephelus merra	NC_022509	17,017	54.8	Miya, *et al.*
Epinephelus moara	NC_017891	16,696	55.0	Liu, *et al.*
Epinephelus quoyans	NC_021450	16,797	56.5	Peng, *et al.*
Epinephelus sexfasciatus	NC_021765	16,786	55.6	Du, *et al.*
Epinephelus stictus	NC_021133	16,524	55.5	Ye, *et al.*
Epinephelus tauvina	NC_056898	16,787	55.0	Liang, *et al.*
Epinephelus trimaculatus	NC_021612	16,761	56.1	Ye, *et al.*
Epinephelus tukula	NC_024039	16,503	54.8	Yang, *et al.*
Grammistes sexlineatus	NC_024108	16,502	52.5	Xu, *et al.*
Hyporthodus haifensis	MW015093	16,525	55.7	Vella, *et al.*
Hyporthodus octofasciatus	NC_020047	16,545	56.0	Zhuang, *et al.*
Hyporthodus septemfasciatus	NC_013829	16,558	55.4	Oh, *et al.*
Plectropomus areolatus	NC_021405	16,770	56.4	Shen, *et al.*
Plectropomus laevis	NC_057260	16,774	56.9	Li, *et al.*
Plectropomus leopardus	NC_008449	16,714	56.8	Zhu, *et al.*
Triso dermopterus	NC_022140	16,605	53.9	Zhuang, *et al.*
Variola albimarginata	NC_022139	16,768	56.1	Zhuang, *et al.*
Variola louti	NC_022138	16,770	56.2	Zhuang, *et al.*
**Outgroups**				
Lates japonicus	NC_034339	16,590	53.2	Satoh, *et al.*
Pagrus major	NC_003196	17,031	54.5	Miya, *et al.*
